# Co-Occurrence of *RAD21* and *TNFAIP3* Mutations in Cornelia de Lange Syndrome with Pustular Psoriasis: Potential Molecular Interactions

**DOI:** 10.3390/ijms262110783

**Published:** 2025-11-06

**Authors:** Beatriz E. Orozco, Cindy V. Orozco, Esperanza Meléndez, María F. Mangones, José Valderrama, Adalberto Lobato, Pilar Garavito-Galofre, Jorge I. Vélez, Oscar M. Vidal

**Affiliations:** 1 Department of Medicine, Universidad del Norte, Barranquilla 081007, Colombia; orozcob@uninorte.edu.co (B.E.O.); cvorozco@uninorte.edu.co (C.V.O.); mmangones@uninorte.edu.co (M.F.M.);; 2 Department of Industrial Engineering, Universidad del Norte, Barranquilla 081007, Colombia; jvelezv@uninorte.edu.co

**Keywords:** Cornelia de Lange syndrome, psoriasis, *RAD21*, *TNAFAIP3*, genetic interplay, cohesin, NF-κB signaling

## Abstract

Cornelia de Lange Syndrome (CdLS) is a rare multisystem developmental disorder caused primarily by mutations in cohesin complex genes, including *RAD21*. Psoriasis is a chronic inflammatory skin disease linked to immune dysregulation, notably involving *TNFAIP3* (*A20*), a negative regulator of NF-κB signaling. Although case reports have suggested a possible coexistence of CdLS and psoriasis, the underlying molecular basis has remained unexplored. Here we report the first case of molecular co-occurrence of CdLS and generalized pustular psoriasis in a patient with novel heterozygous nonsense variant in *RAD21* (c.1306C>T, p.Gln436*), pathogenic for CdLS type 4, and a previously unreported truncating variant in *TNFAIP3* (c.2199C>A, p.Cys733*), predicted to disrupt NF-κB regulation and classified as a variant of uncertain significance. Structural protein modeling showed significant conformational disruption in RAD21 and partial truncation of the ZnF domains of TNFAIP3, supporting their functional impact. This study is the first to suggest a possible molecular mechanism that may explain the rare co-occurrence of CdLS and psoriasis: RAD21 deficiency disrupts chromatin architecture and immune gene regulation, while TNFAIP3 loss-of-function removes critical NF-κB inhibition, resulting in synergistic developmental and inflammatory phenotypes. Secondary transcriptomic data analysis further suggests that *RAD21* knockdown may downregulate TNFAIP3 expression, providing a possible mechanistic intersection. Our findings provide the first molecular evidence linking *RAD21* and *TNFAIP3*, introducing a novel pathogenic hypothesis connecting cohesin dysfunction and immune dysregulation. This work expands the mutational spectrum of both genes and opens a new avenue for understanding developmental-inflammatory disease overlap.

## 1. Introduction

Cornelia de Lange syndrome (CdLS) is a complex congenital disorder originally characterized by the pediatrician Cornelia de Lange in 1933 [1]. The syndrome comprises growth and cognitive retardation, hypertrichosis, and upper-limb reduction defects that range from subtle phalangeal abnormalities to oligodactyly (missing digits), cardiac, ophthalmologic, and genitourinary anomalies, together with distinctive facial features such as fine arched eyebrows, synophrys, long eyelashes, low-set posteriorly rotated ears, long philtrum, thin upper lip, and a depressed nasal bridge with anteverted nares [2]. Its prevalence is estimated to be between 1 in 10,000 to 1 in 30,000 individuals, affecting males and females equally, with most cases occurring sporadically despite familial occurrences documented [3].

The etiology of CdLS indicates that defects in the cohesin pathway are the primary drivers of its clinical manifestations and characteristic features [4]. The cohesin protein complex and its regulatory factors are essential for mitosis, especially for the accurate separation of sister chromatids. Additionally, cohesin plays crucial roles in maintaining genome stability, regulating gene expression, modulating chromatin architecture, and organizing the three-dimensional genome structure [5].

To date, associations between CdLS and mutations in the *NIPBL*, *SMC1A*, *SMC3*, *BRD4*, *HDAC8*, *RAD21*, and *ANKRD* genes have been identified [6]. Although CdLS is a genetic disorder, most cases are sporadic, and result from de novo heterozygous mutations [7]. Frameshift or missense mutations, including truncations that remove RAD21 domains needed to bind SMC1A, compromise cohesin’s effectiveness in stabilizing the cohesin-NIPBL-DNA complex, which weakens chromatin loop formation and enhancer-promoter communication critical for normal morphology, growth, and organ development [8]. Hence, *RAD21* mutations drive the CdLS phenotype primarily by disrupting cohesin’s role in higher-order chromatin structure and developmental gene expression regulation, resulting in the diverse growth, cognitive, limb, cardiac, ophthalmologic, genitourinary, and facial abnormalities characteristic of the syndrome.

Psoriasis is a chronic, immune-mediated inflammatory skin disorder characterized by hyperproliferation of keratinocytes and infiltration of immune cells [9]. *TNFAIP3* encodes A20, a ubiquitin-editing enzyme that is a key negative regulator of the NF-κB signaling pathway, which controls inflammation and immune responses [10]. Loss-of-function or pathogenic variants in *TNFAIP3* lead to dysregulated NF-κB activation, resulting in excessive inflammatory responses and autoimmune/autoinflammatory diseases such as pustular psoriasis. *TNFAIP3* mutations disrupt the negative regulation of NF-κB signaling, leading to uncontrolled inflammatory responses [11]. Such mutations have been implicated in autoinflammatory disorders, including generalized pustular psoriasis (GPP), through enhanced keratinocyte activation and neutrophil recruitment [12]. Alt hough loss-of-function variants in *TNFAIP3* are rare, they can predispose carriers to early-onset and severe psoriasis phenotypes [13,14]. These findings suggest that TNFAIP3 plays a critical role in cutaneous immune homeostasis and that its disruption may act as a molecular trigger for pustular psoriasis.

Although exceedingly rare, few case reports have documented the coexistence of CdLS and GPP or other psoriatic manifestations. Lubkov et al. (2018) reported a 15-year-old girl diagnosed with CdLS who also developed psoriasis [14] and Mugheddu et al. (2020) reported a pediatric case of CdLS coexisting with GPP, hypothesizing a shared inflammatory or genetic predisposition [15,16]. A similar case in a young patient from Pakistan with classical CdLS features and a history of recurring pustular skin eruptions consistent with psoriasis [17]. These cases suggest that although the coexistence of CdLS and GPP is rare, it may reflect a shared molecular basis possibly involving the *RAD21* and *TNFAIP3* genes, which are implicated in both developmental and inflammatory pathways.

*RAD21* is a core component of the cohesin complex, critical for chromatin structure maintenance, gene transcription regulation, and genomic stability [18]. Mutations in *RAD21* are known to cause developmental abnormalities through cohesin dysfunction and transcriptional dysregulation, as seen in CdLS [19]. On the other hand, *TNFAIP3* encodes A20, a key negative regulator of the NF-κB signaling pathway. Mutations in this gene lead to chronic inflammation and autoimmune-like phenotypes due to uncontrolled NF-κB activation [13]. The cohesin complex, including RAD21, plays a role in regulating genes involved in immune response and inflammation. Hence, *RAD21* dysfunction may indirectly influence inflammatory pathways, including NF-κB signaling [20,21].

Here we study the genetics correlation and potential molecular intersection between *RAD21* and *TNFAIP3* mutations that could explain the co-occurrence of these two disorders. While mutations in *RAD21* cause developmental defects via cohesin dysfunction and transcriptional dysregulation and modulate inflammatory pathways indirectly, mutations in *TNFAIP3* lead to immune dysregulation and chronic inflammation through failure to restrain NF-κB signaling [22]. Thus, the combined effect of impaired transcriptional regulation (i.e., a mutation in *RAD21*) and enhanced inflammatory signaling (i.e., a mutation in *TNFAIP3*) may explain the coexistence of developmental abnormalities (CdLS) and inflammatory skin disease (pustular psoriasis) in the same patient [22].

We hypothesize that the combined impact of impaired transcriptional regulation caused by the *RAD21* mutation and increased pro-inflammatory signaling from the *TNFAIP3* variant may contribute to the co-occurrence of CdLS and GPP in this individual, suggesting a possible mechanistic link. This hypothesis suggests a novel pathophysiological link between developmental and inflammatory pathways, mediated through shared transcriptional and epigenetic regulatory mechanisms.

## 2. Results

### 2.1. Proband Phenotype

**Figure 1:**
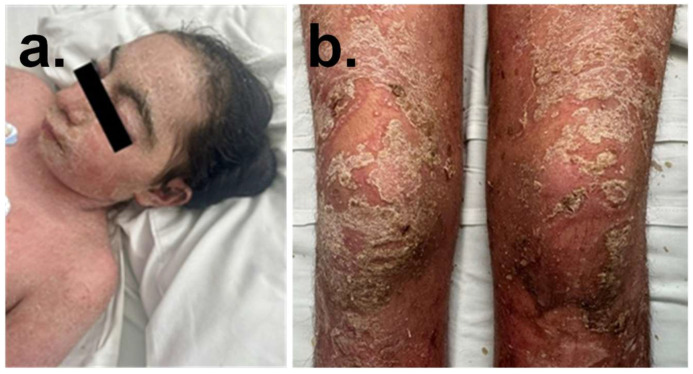
(**a**) Patient’s clinical features with CdLS; synophrys are an important feature of CdLS. (**b**) Multiple pustules on the body head, arms and legs of patient; widespread erythema, covered thick yellowish scales, and circular distributed pustules on the scalp, trunk, perineum, and extremities.

The proband is a 13-year-old female, Colombian, born from a vanishing twin pregnancy, with a maternal history of cleft palate. She presented with an 8-month history of erythematous, scaly, and pruritic plaques initially localized to the periareolar and supraumbilical regions, which progressively extended to the scalp, anterior trunk, and lower extremities, culminating in erythroderma (Figure 1). Physical examination revealed short stature, low weight-for-age, a long face, micrognathia, arched eyebrows with synophrys, hypertrichosis, and upper-limb reduction defects that range from subtle phalangeal abnormalities to oligodactyly (missing digits), mild hirsutism, and hypertelorism in the mammary region. Skin findings included thin, scaly erythematous plaques affecting the scalp, face, trunk, and limbs; ivory-colored circinate lesions with peripheral pustules on the thighs; and coalescent purulent lakes in the bilateral pretibial areas, with associated palmoplantar scaling. Neurocognitive features include dysprosody and limited social interaction. Histopathology confirmed pustular psoriasis, and clinical exome sequencing identified a heterozygous likely pathogenic variant in RAD21, associated with CdLS type 4 (CdLS4), and a heterozygous variant of uncertain significance in TNFAIP3, which is linked to familial autoinflammatory syndrome.

### 2.2. Molecular Characterization

#### 2.2.1. RAD21

The *RAD21* c.1306C>T p.(Gln436*) variant generates a premature stop codon in exon 10 (of 14). It is classified as probably pathogenic based on ACMG/AMP/ClinGen SVI guidelines. Pathogenic variants in the *RAD21* gene are associated with CdLS4 [23,24,25,26]. CdLS is a multisystemic malformation syndrome recognized primarily based on characteristic facial dysmorphism, including a low anterior hairline, arched eyebrows, synophrys, anteverted nostrils, maxillary prognathism, long philtrum, thin lips, and a “pouty” mouth, in association with prenatal and postnatal growth retardation, intellectual disability, and, in many cases, upper limb abnormalities [27]. However, there is wide clinical variability in this disorder, with milder phenotypes that can be difficult to determine based on physical characteristics. *RAD21* has been associated with a milder form of CdLS compared to other genes such as *NIPBL*, *HDAC8*, and *SMC1A*.

#### 2.2.2. TNFAIP3

The *TNFAIP3* variant c.2199C>A, resulting in p.(Cys733*), introduces a premature stop codon in exon 9 (the final exon) of the gene. Numerous truncating, nonsense, frameshift, and splice-site variants in *TNFAIP3* (encoding the A20 protein) have been documented and are known to cause A20 haploinsufficiency (HA20), which manifests as autoinflammatory or autoimmune phenotypes [22]. This variant is novel and has not been previously reported in the literature or among individuals diagnosed with *TNFAIP3*-related disorders. According to the ACMG/AMP guidelines [28] and ClinVar [29], it is currently classified as a variant of uncertain significance. Clinically, affected individuals present with features akin to familial autoinflammatory syndrome resembling Behçet’s disease (OMIM, 616744) [30,31], an autosomal dominant condition characterized by recurrent ulcerations of mucosal surfaces, especially in the oral and genital regions [28], driven by inappropriate activation of inflammatory cytokines [30,32]. Symptom onset typically occurs in the first or second decades of life, with a variable additional manifestation including skin rash, uveitis, and polyarthritis [28,29].

### 2.3. Protein Characterization

#### 2.3.1. Protein Characterization of RAD21

**Figure 2:**
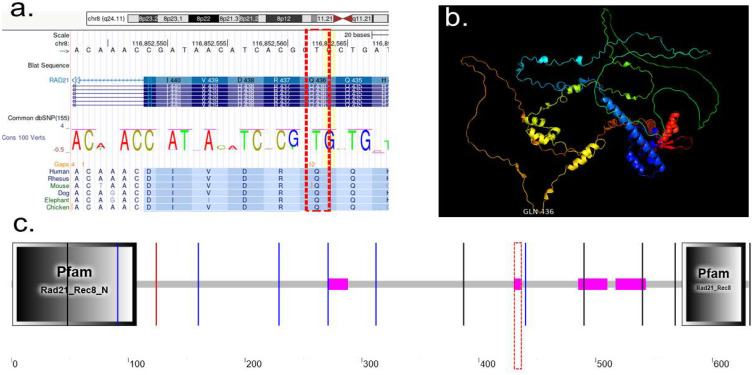
Characterization of a de novo heterozygous *RAD21* mutation identified in the proband. (**a**) UCSC Genome Browser view showing the location of the RAD21 variant NM_006265.2:c.1306C>T, resulting in a premature stop codon at position p.(Gln436*). The mutation occurs on the minus strand in exon 10 of 14, at chromosome 8: position 116,852,561 (GRCh38/hg38). Multispecies alignment shows the high conservation of the affected region across vertebrates. The variant introduces a nonsense mutation, replacing a glutamine residue (Q) with a stop codon, predicted to result in truncated RAD21 protein. (**b**) 3D structure of the RAD21 protein showing the location of the truncated glutamine residue at position 436 (GLN436) in brown, marking the termination point of the protein caused by the mutation. (**c**) Schematic diagram of the RAD21 protein (∼631 amino acids), highlighting conserved domains identified by Pfam. The N-terminal domain (Rad21_Rec8_N) and C-terminal domain (Rad21_Rec8) are marked in black boxes. The red dashed line indicates the location of the nonsense mutation at position 436, within a region of low compositional complexity (amino acids 430–436), potentially impacting protein function and interactions.

Double-strand-break repair protein rad21 homolog, is a member of the cohesin complex, involved in sister chromatid cohesion from the time of DNA replication in S phase to their segregation in mitosis, essential for proper chromosome segregation, post-replicative DNA repair, and the prevention of inappropriate recombination between repetitive regions [33]. Specific mutation in RAD21, NM_006265.2:c.1306C>T p.(Gln436*), Transcript: NM_006265.1 cDNA: c.1306–1308 Strand: -Exon: Exon 10 of 14 located at chr8:10,673,499–10,658,686 (Figure 2a). This variant causes change on the Glycine amino acid with termination codon. RAD21 protein cartoon view at the active loop at the GLN—436 (Figure 2b). Schematic representation of RAD21 and its domains; conserved N-terminal region and conserved C-terminal region found in eukaryotic cohesins of the Rad21, Rec8 and Scc1 families; mutation happens at region of low compositional complexity, starts at position 430 and ends at position 436 (Figure 2c).

#### 2.3.2. Protein Characterization of TNFAIP3

**Figure 3:**
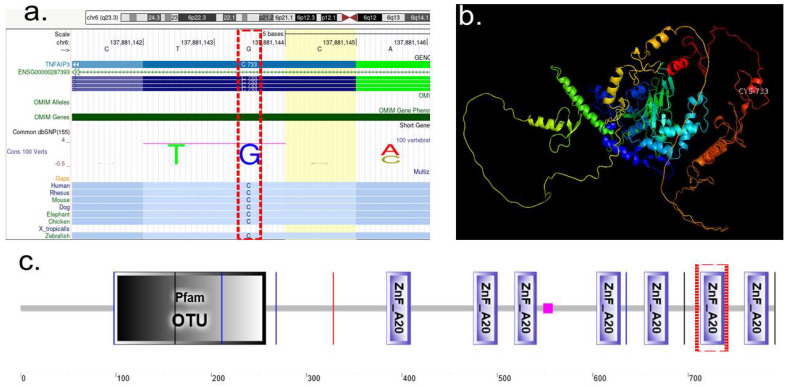
Characterization of a de novo heterozygous *TNFAIP3* nonsense mutation in the proband. (**a**) UCSC Genome Browser view highlighting the TNFAIP3 variant NM_001270507.1:c.2199C>A, which results in a premature stop codon at position p.(Cys733*). This nonsense mutation occurs on the plus strand in exon 9 of 9 at chromosome 6: position 137,881,145 (GRCh38/hg38). Multispecies alignment demonstrates conservation of the affected nucleotide across vertebrates, supporting potential functional importance. The variant leads to substitution of cysteine at position 733 with a termination codon, truncating the protein. (**b**) 3D structure of the TNFAIP3 protein, with the C-terminal truncation site (Cys733) shown in red. The mutation eliminates a significant portion of the C-terminal domain, potentially impacting protein stability or function. (**c**) Schematic diagram of the TNFAIP3 protein (790 amino acids total), showing the conserved OTU (ovarian tumor) domain at the N-terminus and multiple C-terminal zinc finger (ZnF_A20) domains. The truncation at amino acid 733 (highlighted by a red dashed line) disrupts the final ZnF_A20 domain, removing the last 57 amino acids, which may impair the protein’s regulatory role in ubiquitin editing and NF-κB signaling.

Tumor necrosis factor alpha-induced protein 3 (TNFAIP3), ubiquitin-editing enzyme that contains both ubiquitin ligase and deubiquitinase activities. Involved in immune and inflammatory responses signaled by cytokines, such as TNF-alpha and IL-1 beta, or pathogens via Toll-like receptors (TLRs) through terminating NF-kappa-B activity [34,35]. The A20-type zinc fingers mediate the ubiquitin ligase activity. The A20-type zinc finger 4 selectively recognizes ‘Lys-63’-linked polyubiquitin. The A20-type zinc finger 4–7 are sufficient to bind polyubiquitin, the OTU domain mediates the deubiquitinase activity (Figure 3c) [36]. TNFAIP3 mutation described as, TNFAIP3 NM_001270507.1:c.2199 C>A. p.(Cys733*), Transcript: Transcript: ENST00000612899.5, cDNA: c.2197–2199, Strand: +, Exon: Exon 9 of 9. This variant causes change on the Glycine amino acid with termination codon Cys733* (Figure 3a). TNFAIP3 protein cartoon shows the mutation site active loop at the CYS733 (Figure 3b). Figure 3c shows, Schematic representation of TNFAIP3, truncation site at amino acid 733 of 790, this variant removes the final 57 amino acids. See colored bar dash-dotted line, this is a region of ZnF_A20 domain, starts at position At amino acid 733 of 790.

### 2.4. Mutational Analysis of RAD21 and TNFAIP3

**Figure 4:**
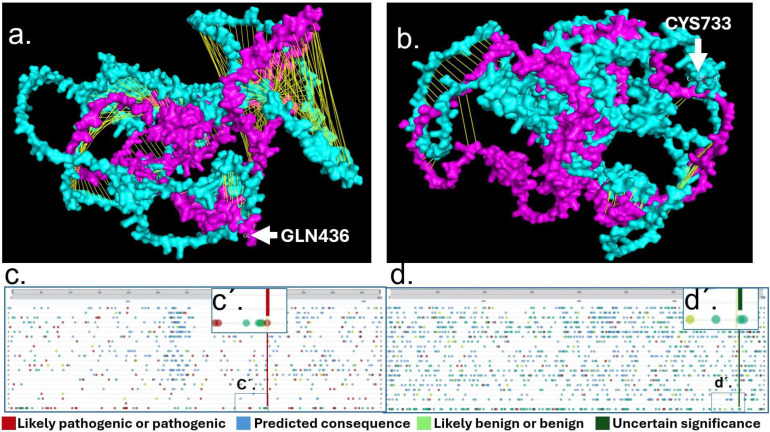
Structural and variant interpretation analyses of *RAD21* and *TNFAIP3* truncating mutations. (**a**) Structural alignment of RAD21 wild-type (WT, cyan) versus the p.(Gln436*) truncating mutant (magenta), highlighting disrupted alignment due to conformational changes introduced by the premature stop codon. Misaligned regions are shown by yellow connecting lines, indicating a substantial structural deviation in the mutant. (**b**) Structural alignment of TNFAIP3 wild-type (WT, cyan) versus the p.(Cys733*) truncating mutant (magenta), showing comparatively less structural disruption than observed in RAD21. Yellow lines represent alignment mismatches, indicating a relatively more conserved structure despite the truncation. (**c**) *RAD21* variant viewer from the UniProt database displaying reported variants along the protein sequence. A red line highlights the p.(Gln436*) variant, classified as likely pathogenic. (**c’**) Zoomed-in view showing the specific pathogenic classification of Gln436 (GLN436) in red. (**d**) *TNFAIP3* variant viewer from UniProt, showing the distribution of known variants across the protein. The p.(Cys733*) variant is indicated by a green line, suggesting uncertain clinical significance. (**d’**) Zoomed-in view highlights the position of Cys733 (CYS733) in green, corresponding to a variant of uncertain significance.

We applied AlphaFold Protein Structure Database (AFDB) to obtain RAD21 and TNFAIP3 WT protein structures [37], and AlphaFold 3, to study the WT protein (cyan color) against the altered proteins RAD21 Mutant p. (Gln436*) and TNFAIP3 Mutant p.(Cys733*)(magenta). RAD21 WT vs. RAD21 Mutant p. (Gln436*), alignment analysis showed significant disruption visible by yellow lines due to mutant conformational changes in RAD21 protein (Figure 4a). Alignment analysis of TNFAIP3 WT vs. TNFAIP3 Mutant p.(Cys733*) showed comparatively a less disrupted alignment assed by yellow lines within the two molecules (Figure 4b). Variant Viewer from UniProt showed RAD21 with a red line likely pathogenic or pathogenic (Figure 4c) showing GLN436. TNFAIP3 variant viewer showed CYS 733 in green line as a variant of uncertain significance (Figure 4d).

### 2.5. Mutational Analysis of RAD21 and TNFAIP3

To investigate the potential interaction between RAD21, a core subunit of the cohesin complex, and TNFAIP3 (also known as A20), a ubiquitin-editing enzyme involved in NF-κB signaling, we explored multiple curated protein–protein interaction (PPI) databases and published interactome studies, including comprehensive resources such as STRING, BioGRID, IntAct, and high-throughput mass spectrometry-based interactomes, as well as targeted literature reviews [38]. RAD21 is well-documented to interact with canonical cohesin complex components, including SMC1A, SMC3, STAG1/2, PDS5A/B, WAPL, and NIPBL, as well as regulatory factors involved in chromosomal cohesion, DNA damage response, and transcriptional regulation [39,40]. Similarly, the TNFAIP3 interactome has been shown to include key mediators of inflammatory and immune signaling, such as TRAF2, TRAF6, IKKγ (NEMO), TAX1BP1, TNIP1, and 14-3-3 proteins, as well as components of autophagy machinery including ATG16L1 [41,42] (see Supplementary Figure S1). Furthermore, studies using from yeast two-hybrid screening and affinity purification–mass spectrometry experiments [43], no direct or indirect interaction between RAD21 and TNFAIP3/A20 has been identified. This observation remained consistent across all major curated interaction repositories, including STRING (v12.0), BioGRID (v4.4), and IntAct (EMBL-EBI). A recent large-scale human interactome mapping study also failed to detect any physical association between these proteins [43].

A patient carrying *RAD21* and *TNFAIP3* variants could experience dysregulated amplitude and duration of NF-κB-dependent transcription: altered chromatin wiring of inflammatory genes (RAD21) plus impaired signal shut-off (A20). That combination is biologically coherent with severe skin inflammation such as pustular psoriasis, even without a direct RAD21–A20 physical interaction.

**Figure 5:**
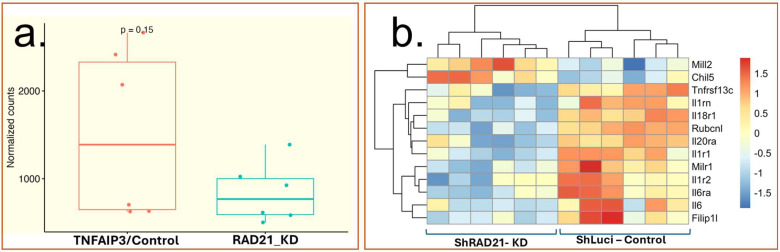
(**a**) Boxplot showing normalized TNFAIP3 expression levels in control cells (shLuciferase) versus RAD21 knockdown cells (shRAD21). TNFAIP3 expression is reduced in RAD21-depleted cells, suggesting a regulatory relationship between RAD21 and TNFAIP3 transcription. (**b**) Heatmap displaying differential expression of inflammation- and immune-related genes following RAD21 knockdown. Genes shown are clustered based on expression patterns, with many pro-inflammatory genes upregulated upon RAD21 depletion, indicating that RAD21 may play a role in repressing inflammatory gene expression.

We performed secondary data analysis from the Gene Expression Omnibus (GEO) data set GSE110440, which investigated mRNA expression patterns by RAD21 knockdown in hematopoietic stem cells (HSPCs) [44]. RAD21 knockdown is associated with a trend toward reduced TNFAIP3 mRNA expression compared to control (Figure 5a), although this difference did not reach statistical significance. Additionally, heatmap visualization highlighted altered expression of a subset of inflammatory and immune system genes subject to RAD21 regulation (Figure 5b).

## 3. Discussion

The 13-year-old female was characterized as a vanishing twin pregnancy. Individuals with Cornelia de Lange syndrome (CdLS) originating from a vanishing twin syndrome (VTS) may exhibit compounded developmental alterations due to disruptions in embryological environments. CdLS is characterized by mutations in cohesin-pathway genes, which are crucial for regulating chromatin structure and gene expression during neurogenesis [4]. Additionally, VTS has been associated to localized vascular or inflammatory changes within the environment of the surviving twin [45]. In this case, the clinical features of patient with CdLS phenotype correlated with RAD21 gene alterations, while TNFAIP3 modifications were consistent with her history of erythematous, scaly, and pruritic plaques, culminating in erythroderma (Figure 1).

CdLS and generalized pustular psoriasis (GPP) are distinct conditions with different genetic roots and clinical manifestations. CdLS is a rare disorder due to heterozygous mutations in cohesin complex genes like *RAD21*, affecting developmental pathways [46] and typically not overlapping with inflammatory skin diseases. GPP, meanwhile, is characterized by immune dysregulation, where *TNFAIP3* plays a key role [47,48,49,50].

The co-occurrence in one individual is extremely rare and has only been reported in a handful of cases worldwide, making the present case highly significant for both clinicians and researchers [14,51]. Recent molecular studies have identified potential interactions between developmental and inflammatory pathways, such as cohesin-mediated regulation of NF-κB target genes and inflammatory cytokine profiles [21,44,52]. This overlap is just beginning to be explored in the scientific literature and suggests previously unrecognized genetic and pathway connections [53].

A premature stop codon in exon 10 of 14 the *RAD2* gene, corresponding to the c.1306C>T p.(Gln436*) variant, has been reported as pathogenic in association with Cornelia Lange syndrome type 4 (CdLS4) according to ClinVar [54]. However, no peer-reviewed publications have been cited in the ClinVar entry describing individuals harboring this specific variant. The TNFAIP3 variant c.2199C>A p.(Cys733*) introduces a premature stop codon in exon 9 of 9 and similarly has not been documented in published literature or in ClinVar Miner listings for TNFAIP3 [11]. Notably, an extensive catalog of truncating mutations in the TNFAIP3 (A20) gene, including nonsense, frameshift, and splice-site variants, has been reported in patients with haploinsufficiency of A20 (HA20), a dominantly inherited autoinflammatory and autoimmune condition. Zhou et al. (2016) first identified several heterozygous truncating mutations across the N-terminal OTU and C-terminal zinc finger domains of A20, which lead to early-onset systemic inflammation via haploinsufficiency rather than dominant-negative effects [11]. Subsequent studies have reinforced this finding: the majority of HA20-associated variants are truncating mutations, while pathogenic missense variants are rare and tipically require functional analyses for validation [55]. A recent Japanese cohort study further confirmed that frameshift, nonsense, and splice-site variants impair the inhibitory effect of A20 on NF-κB signaling, as demonstrated by their reduced ability to suppress NF-κB-driven reporter activity [56].

Characterization of the RAD21 protein revealed a substitution of glycine with a termination codon, resulting in truncated protein synthesis. Analysis demonstrated that the active protein loop is positioned at Gln-436, with a low-complexity region spanning residues 430–436 (Figure 2). While pathogenic variants in *RAD21* are associated with CdLS primarly cluster within well-characterized functional domains, such as the SMC3-binding (aa 1–103), STAG1/2-binding (aa 362–403), and SMC1A-binding regions (aa 558–628) [57], the functional significance of less-characterized protein regions remains largely unexplored. The c.1306C>T (p.Gln436*) variant introduces a premature stop codon at position 436, truncating the protein immediately following this low-complexity segment. The resulting RAD21 molecule (UniProt: Q99215, Cohesin complex component) is missing ~195 amino acids from the C-terminal domain, which is essential for STAG1/2 binding and for facilitating interactions with other cohesin subunits. This truncation severely disrupts RAD21’s ability to stably integrate into the cohesin complex, hence impairing sister chromatid cohesion and compromising cellular DNA double-strand break repair mechanisms [39,40].

Characterization of the TNFAIP3 protein identified the truncation site at amino acid 733, which affects the C-terminal part of the zinc finger 6 and 7 (ZnF 6–7) domains, approximately spanning aa 701–775. This disruption Likely abolishes or severely impairs A20’s capacity to bind polyubiquitin chains, a function critical for terminating inflammatory responses. Loss of ZnF 7 has been shown to impair A20’s inhibitory function on NF-κB, resulting in dysregulated inflammatory responses [58]. The p.Cys733* variant results in thus introduces a premature stop codon, leading to truncation of the distal proportion of the A20 protein, including the essential region of ZnF 7 required for suppressing NF-κB signaling. Given its location in the terminal exon, the altered transcript may evade nonsense-mediated decay (NMD), and produce a truncated but nonfunctional protein, consistent with a dominant loss-of-function mechanism seen in HA20 [11].

Structural modeling with the AlphaFold Protein Structure Database (AFDB) revealed significant disruption in the predicted conformation of the RAD21 mutant protein (Figure 4a), whereas TNFAIP3 mutant protein displayed comparatively less pronounced misalignment (Figure 4b). Analysis using the UniProt Variant Viewer further supported these observations: the *RAD21* variant as likely pathogenic or pathogenic, while *TNFAIP3* variant was classified as a variant of uncertain significance (Figure 4c).

To explore the potential interaction between RAD21 and TNFAIP3, we performed secondary data analysis from the Gene Expression Omnibus (GEO) dataset GSE110440 [44]. In this analysis, we compared the gene expression levels of TNFAIP3 in control vs. RAD21 knockdown conditions. TNFAIP3 expression was reduced in the RAD21 knockdown group relative to controls, although this reduction did not achieve statistical significance (*p* = 0.15). The observed log_2_ fold change of approximately −0.85 indicates an almost two-fold decrease in expression, suggesting a trend toward downregulation. While not statistically conclusive, this trend may nonetheless reflect a biologically relevant influence of RAD21 depletion on TNFAIP3 regulation, warranting further investigation with larger sample sizes or complementary experimental strategies.

**Figure 6:**
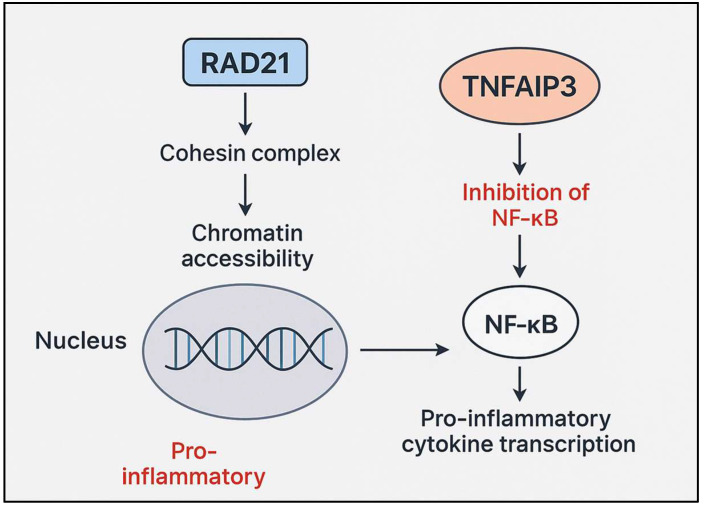
Proposed model of the molecular interaction between RAD21 and TNFAIP3 in regulating inflammation.

Although *RAD21* and *TNFAIP3* (A20) operate at distinct regulatory levels, mutations in these genes may converge functionally on the TNFα/NF-κB signaling axis, potentially amplifying inflammatory dysregulation. RAD21, a core component of the cohesin complex, facilitates stimulus-inducible gene expression by enabling enhancer–promoter communication at NF-κB-responsive loci during inflammatory signaling. Impaired cohesion function, as observed in CdLS, can attenuate or misalign transcriptional responses, resulting in either insufficient or aberrant gene activation [59,60,61]. By contrast, *TNFAIP3* encodes the feedback inhibitor A20, which terminates NF-κB signaling via deubiquitination of key intermediates such as RIPK1 and TRAF6 [62,63]. Heterozygous loss-of-function mutations in *TNFAIP3*, as observed in haploinsufficiency of A20 (HA20), removes this critical brake, prolonging NF-κB activation. In a patient harboring both a *RAD21* nonsense mutation (CdLS4) and a *TNFAIP3* truncation, these dual defects could synergistically exacerbate inflammation: RAD21 deficiency may blunt the precision of NF-κB-driven transcriptional initiation, while A20 haploinsufficiency removes negative feedback, resulting in a pro-inflammatory gene expression program that is both misregulated and persistent. This pathway-level convergence, depicted in Figure 6, may underlie complex inflammatory phenotypes such, as early-onset pustular psoriasis or autoinflammatory disease, in affected individuals.

*RAD21* is a core component of the cohesin complex, which plays a pivotal role in regulating genome architecture by mediating chromatin looping and organizing topologically associated domains (TADs). Such organization is essential for precise enhancer-promoter interactions and for broader transcriptional programs in development and immune responses [18,21,64,65]. Deficiency of RAD21 or disruption of cohesin-mediated architecture can loosen chromatin interactions, leading to altered transcriptional activity—sometimes activation or misregulation of immune relevant genes [44,66,67,68]. In the immune system, RAD21 dysfunction alters the accessibility of NF-κB target genes, influencing inflammatory signaling pathways [21,44]. In parallel, *TNFAIP3* (A20) encodes a repressor of NF-κB and inflammatory signals [69], and its expression is subject to epigenetic regulation and histone modification [70], with potential crosstalk to chromatin remodeling mechanisms. Reduced H3K4me3 methylation at the TNFAIP3 promoter region correlates with decreased expression and heightened inflammation in autoimmune conditions [70,71]. By extension, loss of RAD21 and cohesin function could disrupt chromatin looping at immune loci including TNFAIP3, either by impairing enhancer-promoter interactions or by altering epigenetic landscapes required for proper gene activation or repression [66,67,72]. Although research in this area is still developing, current evidence strongly suggests that developmental chromatin regulators like *RAD21*/cohesin can influence immune gene transcription, including *TNFAIP3*, through both direct and indirect mechanisms. The balance between RAD21-mediated chromatin remodeling and TNFAIP3-mediated NF-κB inhibition plays a critical role in modulating inflammatory responses.

Growing evidence shows that *RAD21* and *TNFAIP3* variants may play a role in other unexplained inflammatory or developmental syndromes. *RAD21* mutations are implicated in a spectrum of cohesinopathies beyond CdLS, often presenting with developmental delay, multiorgan involvement, and sometimes immune dysregulation [21,73,74]. Recent studies also show that *RAD21* can modulate chromatin accessibility at enhancer regions for immune genes, suggesting a plausible mechanism for broader inflammatory phenotypes [21]. Pathogenic variants in *TNFAIP3* (A20), on the other hand, cause autoinflammatory diseases such as A20 haploinsufficiency (HA20), which can present diverse autoimmune or inflammatory symptoms that may overlap with or mimic other syndromes [13,75,76,77]. Given that clinical presentations associated with *RAD21* and *TNFAIP3* variants are broad and can overlap with other unexplained genetic syndromes, considering these genes in cases of unclear developmental or inflammatory disease may help identify underlying molecular etiologies and guide diagnosis or tailored therapies [21,78].

We recognize that single-patient case studies have inherent limitations, such as restricted generalizability and inability to establish definite causal relationships [79]. However, case reports remain crucial in rare disease research, offering valuable opportunities for hypothesis generation, novel disease characterization, and guiding future investigations [80,81,82]. Our work should therefore be viewed as an initial step to open new avenues for investigation and hypothesis generation regarding overlapping developmental and inflammatory disorders, rather than providing definitive evidence [83]. This approach supports the broader tradition of advancing medical science through careful observation and reporting of individual, unique cases.

Recent findings indicate an interplay between host genetics and microbiome composition in the pathogenesis of psoriasis. For instance, microbial dysbiosis can trigger or worsen inflammation, especially in genetically vulnerable individuals [84]. *RAD21* and *TNFAIP3* variants may compromise skin barrier and immune balance, promoting dysbiosis and, in turn, amplifying NF-κB-driven inflammation [85]. Such dysbiosis could in turn enhance innate and adaptive immune activation, amplifying NF-κB signaling and inflammatory cascades, particularly in genetically sensitized individuals. Futures studies profiling the microbiome in patients with such genetic backgrounds could clarify whether microbial shifts work together with these defects to increase psoriasis severity and inform personalized therapies.

In summary, our findings contribute important evidence to this emerging field by providing genetic, transcriptomic, and structural data on both *RAD21* and *TNFAIP3* variants in a single patient, highlighting a possible molecular link between developmental disorders and immune dysregulation. This advances our understanding of disease overlap and may inform future diagnosis and therapy for complex syndromic presentations. Although our secondary data analysis of GEO series GSE110440 did not show a statistically significant reduction in TNFAIP3 expression upon RAD21 knockdown, the observed trend toward downregulation suggests that cohesin function may influence the transcriptional control of this critical immune regulator. When considered together with the well-established role of A20 as a negative feedback inhibitor of NF-κB, these observations raise the possibility that concurrent RAD21 dysfunction and TNFAIP3 haploinsufficiency could act synergistically to promote dysregulated, prolonged inflammatory responses. This dual genetic disruption provides a plausible molecular framework for understanding the co-occurrence of developmental syndromes such as CdLS and autoinflammatory conditions including psoriasis. Future studies with larger cohorts and functional assays are warranted to validate this interaction and further delineate how cohesin-mediated chromatin architecture interfaces with ubiquitin-mediated feedback regulation to shape immune homeostasis.

## 4. Materials and Methods

### 4.1. Proband Phenotype

A 13-year-old female, presented with an 8-month history of erythematous-squamous pruritic plaques, initially located in the periareolar and supraumbilical regions. The lesions progressively extended to the scalp, anterior trunk, and lower extremities, culminating in erythroderma. She was hospitalized at a local medical center, where a skin biopsy revealed findings compatible with pemphigus foliaceus.

Physical examination: The patient exhibited short stature for age, underweight status, elongated facies, micrognathia, arched eyebrows with synophrys, mild hirsutism, and hypertelorism of the nipples. Based on the constellation of findings, the initial clinical impression included pustular psoriasis versus DITRA syndrome, associated with Cornelia de Lange syndrome. Skin biopsies and a clinical exome were requested.

Histopathology: The biopsy demonstrated regular psoriasiform acanthosis of the epidermis, minimal thinning of suprapapillary plates, neutrophil collections within the stratum corneum forming subcorneal pustules, and a mild perivascular and interstitial lymphocytic infiltrate. These findings confirmed the diagnosis of pustular psoriasis.

### 4.2. Molecular Characterization

To study the co-occurrence of CdLS with Pustular Psoriasis we characterized the genomic alterations of *RAD21* and *TNFAIP3* genes:

Genomic Sequencing and Variant Analysis: Genomic DNA was enzymatically fragmented, and target regions were enriched using DNA capture probes. These regions comprised approximately 41 Mb of the human coding exome (covering >98% of the human genome build GRCh37/hg19 coding RefSeq) as well as the mitochondrial genome. The resulting library was sequenced on an Illumina platform, achieving a minimum depth of 20× across >98% of targeted bases.

Bioinformatic processing included alignment of sequence reads to the GRCh37/hg19 human genome assembly and the revised Cambridge Reference Sequence (rCRS; NC_012920) for mitochondrial DNA. Variant calling, annotation, and extensive filtering were performed. Variants with a minor allele frequency (MAF) < 1% in gnomAD, as well as disease-causing variants reported in Human Gene Mutation Database (HGMD^®^, URL: http://www.hgmd.cf.ac.uk/, accessed on 17 September 2025) [86] and ClinVar [29], were systematically evaluated.

Analysis focused on coding exons and ±10 intronic nucleotides of genes with well-established gene–phenotype correlations [30,31]. All inheritance patterns were considered. Family history and clinical information were incorporated to evaluate the pathogenicity and disease relevance of identified variants. Variants were classified into five categories—pathogenic, likely pathogenic, variant of uncertain significance (VUS), likely benign, and benign—according to ACMG/AMP guidelines [55] recommendations [28], supplemented by ClinGen [87].

Strict quality control and validation procedures were applied. Variants with low sequencing quality or unclear zygosity were confirmed by orthogonal methods, ensuring >99.9% specificity for all reported variants. Mitochondrial variants were reported when heteroplasmy levels were ≥15%. Copy-number variant (CNV) detection software was applied with a sensitivity > 95%. Screening for uniparental disomy (UPD) was conducted using a dedicated algorithm for clinically relevant chromosomal regions (6q24, 7, 11p15.5, 14q32, 15q11q13, 20q13, and 20).

### 4.3. Protein Characterization

The genomic locations of RAD21 and TNFAIP3 were identified using the UCSC Genome Browser (GRCh37/hg19 assembly). Protein sequence and structural information were retrieved from UniProt for RAD21 (UniProt ID: O60216, RAD21_HUMAN) and TNFAIP3 (UniProt ID: P21580, TNAP3_HUMAN) [88]. The three-dimensional protein structures were visualized and rendered using PyMOL version 1.8 (Schrödinger, LLC, New York, NY, USA; https://pymol.org, accessed on 17 September 2025). Domain composition and functional motifs were analyzed through the Simple Modular Architecture Research Tool (SMART) database [89], allowing the identification of conserved regions as well as domains affected by pathogenic variants in both proteins.

### 4.4. Protein Mutational Analysis

Structural analyses of RAD21 and TNFAIP3 proteins were performed using the AlphaFold Protein Structure Database (AFDB) and Alphafold server prediction [90] to retrieve the wild-type (WT) protein models. In addition, AlphaFold 3 was applied to generate mutant structures corresponding to RAD21 p.(Gln436*) and TNFAIP3 p.(Cys733*) [91]. Protein visualization, structural alignment, and comparative analyses were carried out using PyMOL version 1.8 (Schrödinger, LLC; https://pymol.org). Structural alignment of WT (cyan) and mutant (magenta) proteins was performed to assess mutation-induced conformational changes. RAD21 WT versus RAD21 p.(Gln436*) and, TNFAIP3 WT versus TNFAIP3 p.(Cys733*) (Figure 3 and Figure 4).

To complement the structural assessment, UniProt Variant Viewer was used to annotate clinical variant relevance [88]. RAD21 p.(Gln436*) was annotated as likely pathogenic/pathogenic (red indicator, Figure 4c), while TNFAIP3 p.(Cys733*) was classified as a variant of uncertain significance (green indicator, Figure 4d).

To explore functional relevance, pathway annotations were integrated from Reactome [92] and KEGG databases [93] for pathway enrichment analysis was performed using Reactome with RAD21 mapped to cohesin complex-mediated chromosomal segregation and DNA repair pathways, and TNFAIP3 mapped to NF-κB signaling and immune regulatory pathways. Comparative analysis highlighted potential crosstalk between chromatin regulation and immune signaling mediated by these two proteins (see Supplementary Figure S1).

#### Secondary mRNA Expression Analysis

To investigate the potential interaction between RAD21 and TNFAIP3, we performed a secondary analysis of publicly available transcriptomic data from the Gene Expression Omnibus (GEO) database [94]. Specifically, we analyzed series GSE110440 (https://www.ncbi.nlm.nih.gov/geo/query/acc.cgi?acc=GSE11 accessed on 10 September 2025), which contains gene expression profiles following RAD21 knockdown compared with control samples. Data preprocessing, normalization, and comparative statistical analysis were carried out in R [95]. Normalized expression values for TNFAIP3 were compared between conditions using two-sample *t*-test (Pathway enrichment and visualization were carried out using R version 4.4.0 (94); detailed scripts are available in the Supplementary Material).

## 5. Conclusions

Our study provides the first molecular description of the co-occurrence of Cornelia de Lange syndrome (CdLS) and generalized pustular psoriasis (GPP), highlighting the potential mechanistic convergence of *RAD21* and *TNFAIP3* mutations. Although protein–protein interaction database analyses did not reveal a direct interaction between RAD21 and TNFAIP3, secondary expression analyses suggested a trend toward reduced TNFAIP3 expression upon RAD21 knockdown, raising the possibility of indirect regulatory crosstalk. Taken together, our findings raise the possibility that RAD21 dysfunction on developmental transcriptional networks and TNFAIP3 haploinsufficiency on inflammatory regulation could act in parallel to contribute to a dual phenotype encompassing congenital malformations and severe cutaneous autoinflammation. These suggest, but do not prove, a novel developmental–immunological interface mediated by cohesin and NF-κB signaling pathways that broaden the clinical spectrum of CdLS. Future studies utilizing larger patient cohorts, functional genomics, and mechanistic assays will be critical to validate this proposed link and to clarify whether RAD21–TNFAIP3 convergence represents a broader pathogenic axis in rare disease and autoinflammatory conditions.

## Data Availability

The data generated in this study are available from the corresponding author.

## Supplementary Materials

The following supporting information can be downloaded at: https://www.mdpi.com/article/10.3390/ijms262110783/s1. References [95,96,97] has been mentioned in text.

## Abbreviations

The following abbreviations are used in this manuscript:

ACMG	American College of Medical Genetics
AMP	Association for Molecular Pathology
ANKRD	Ankyrin Repeat Domain protein
BRD4	Bromodomain-containing protein 4
CdLS	Cornelia de Lange Syndrome
CNV	Copy-number variant
GEO	Gene Expression Omnibus
GPP	Generalized Pustular Psoriasis
HDAC8	Histone Deacetylase 8
HGMD	Human Gene Mutation Database
HSPCs	Hematopoietic Stem and Progenitor Cells
IL-1	Interleukin 1
NF-κB	Nuclear Factor kappa-light-chain-enhancer of activated B cells
NIPBL	Nipped-B-like protein
NMD	Nonsense-Mediated Decay
OMIM	Online Mendelian Inheritance in Man
OTU	Ovarian tumor domain
RAD21	Double-strand-break repair protein rad21 homolog
SMC1A	Structural Maintenance of Chromosomes 1A
SMC3	Structural Maintenance of Chromosomes 3
TLRs	Toll-like receptors
TNFAIP3	Tumor necrosis factor alpha-induced protein 3 (A20)
VTS	Vanishing twin syndrome
VUS	Variant of Uncertain Significance
ZnF	Zinc finger domain
