# Supplementary material for: Co-Occurrence of RAD21 and TNFAIP3 Mutations in Cornelia de Lange Syndrome with Pustular Psoriasis: Potential Molecular Interactions
Source: Int J Mol Sci. 2025 Nov 6;26(21):10783. doi: 10.3390/ijms262110783 (PMC12609602; doi:10.3390/ijms262110783)
Supplement: Supplementary file 1 [file ijms-26-10783-s001.zip › ijms-3908880-supplementary.pdf]

Supplementary Material

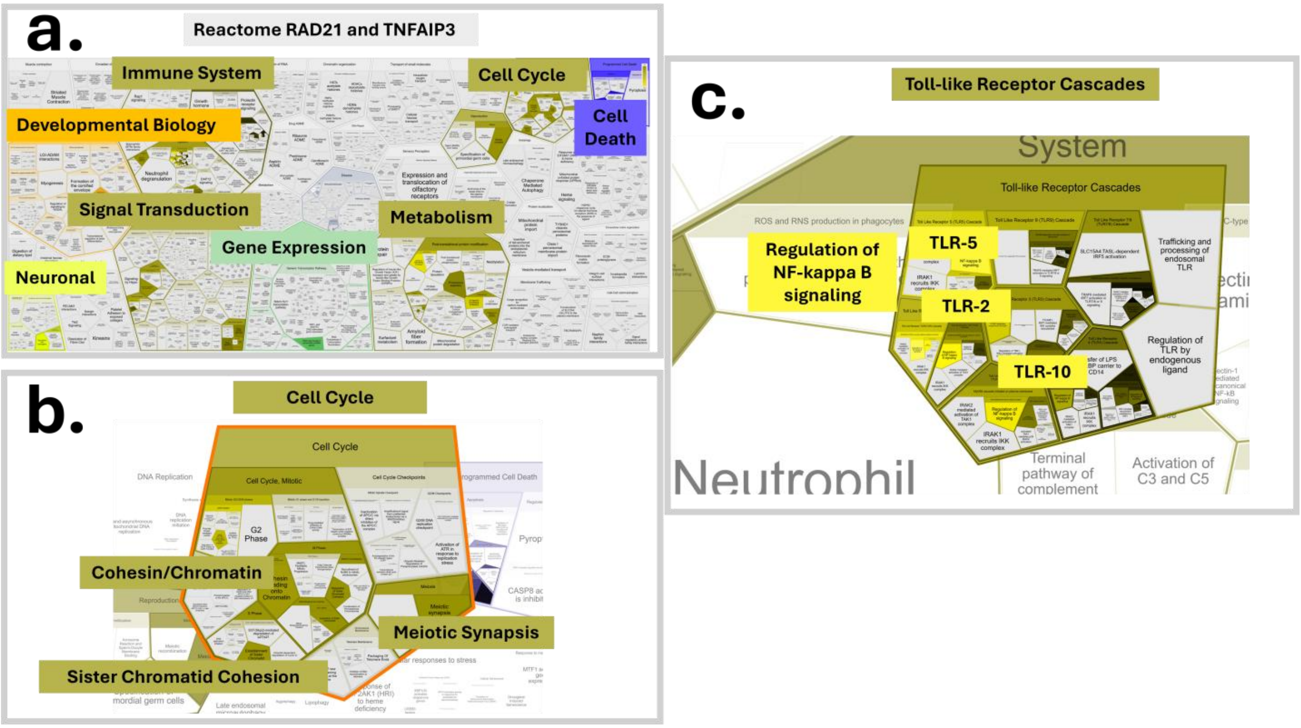

Figure S1. Supplementary material. (a) Whole-reactome analysis of RAD21 and TNFAIP3 molecular interactions, highlighting en- riched pathways including immune system, cell cycle, metabolism, developmental biology, signal transduction, programmed cell death, and neuronal processes. (b) The cell cycle emerged as one of the principal pathways associated with RAD21 and TNFAIP3, with specific enrichment in cohesin/chromatin regulation, sister chromatid cohesion, and meiotic synapsis. (c) The immune system represents the second major pathway, particularly within the toll-like receptor (TLR) cascades, showing modulation of NF-κB sig- naling through TLR2, TLR5, and TLR10. Pathway enrichment analysis was performed using the Reactome Pathway Knowledgebase (Gillespie et al., 2022). .

Supplementary Methods: Expression Analysis of TNFAIP3 under RAD21 Knockdown

1. Data acquisition and preprocessing

Raw RNA-seq count data for hematopoietic stem and progenitor cells (HSPCs) were obtained from the **NCBI Gene Expression Omnibus (GEO)** (accession number **GSE110440**). Raw count data (GSE110440_raw_counts.txt.gz) were imported into **R** (v4.x; R Core Team, 2023) and preprocessed using the **DESeq2** package (Love et al., 2014). Genes with total counts ≤ 10 across all samples were excluded.

R

```
counts <- read.delim(gzfile("GSE110440_raw_counts.txt.gz"),
                     row.names = 1, check.names = FALSE)
```

```
dds <- DESeqDataSetFromMatrix(countData = counts,
                              colData = metadata,
                              design = ~ condition)

dds <- dds[rowSums(counts(dds)) > 10, ]

dds <- DESeq(dds)
```

2. Differential expression analysis

Differential gene expression between **RAD21 knockdown (shRad21)** and **control (shLuci)** samples was assessed with **DESeq2**. Con-

trasts were extracted using:

R

```
res <- results(dds, contrast = c("condition", "RAD21_KD", "Control"))
```

Gene annotations were added using **org.Mm.eg.db** (Carlson, 2023) to convert Ensembl IDs to gene symbols. 41

3. Extraction and analysis of Tnfaip3

The target gene **Tnfaip3** (Ensembl ID: *ENSMUSG00000019850*) was retrieved from the DESeq2 results. Log_2 fold change (LFC), p-

value, and adjusted p-value (Benjamini–Hochberg) were extracted. Normalized expression values were computed as:

R

```
norm_counts <- counts(dds, normalized = TRUE)

tnfaip3_counts <- norm_counts["ENSMUSG00000019850", ]
```

4. Statistical testing and visualization

Normalized **Tnfaip3** expression values were compared between groups. Statistical tests included:

- **Wilcoxon rank-sum test** (via ggpubr::stat_compare_means)
- **Two-tailed Welch's t-test**:

R

```
t.test(expression ~ condition, data = df_plot, alternative = "two.sided")
```

Visualization was performed using **ggplot2** (Wickham, 2016) and **ggpubr**:

R

```
ggboxplot(df_plot, x = "condition", y = "expression",
          color = "condition", add = "jitter",
          ylab = "Normalized counts",
title = "Normalized expression of Tnfaip3") +
stat_compare_means(method = "t.test", label = "p.format")
```
